# FGF20 promotes spinal cord injury repair by inhibiting the formation of necrotic corpuscle P‐MLKL/P‐RIP1/P‐RIP3 in neurons

**DOI:** 10.1111/jcmm.70109

**Published:** 2024-12-16

**Authors:** Xiong Cai, Zhenwen Xie, Juan Zhao, Wenjie Lu, Zhongwei Zhu, Min Chen, Zhiyang Huang, Yibo Ying, Yining Fu, Jiake Xu, Sipin Zhu

**Affiliations:** ^1^ Department of Orthopaedics The Second Affiliated Hospital and Yuying Children's Hospital of Wenzhou Medical University Wenzhou Zhejiang China; ^2^ The First Clinical School of Wenzhou Medical University Wenzhou Zhejiang China; ^3^ Shenzhen Institute of Advanced Technology Chinese Academy of Sciences Shenzhen Guangdong China

**Keywords:** fibroblast growth factor 20, necroptosis, neuroprotection, regeneration, spinal cord injury

## Abstract

The disruption of the local microenvironment subsequent to spinal cord injury (SCI) leads to a substantial loss of neurons in the affected region, which is a major contributing factor to impaired motor function recovery in patients. Fibroblast growth factor 20 (FGF20) is a neurotrophic factor that plays a crucial role in neuronal development and homeostasis. In this study, the recombinant human FGF20 (rhFGF20) was found to mitigate the process of necroptosis in a mouse model of SCI, thereby reducing neural functional deficits and promoting SCI repair. FGF20 protein was injected into the SCI mice via intraperitoneal injection. Using the BMS scale and inclined plane test, we found that FGF20 significantly promoted the recovery of motor function. The Nissl staining revealed the level of neuronal survival within the region of injury. The expression changes of NeuN, GAP43, NF200 and GFAP indicated that FGF20 has the nerve repair ability to delay the formation of glial scar. Through fluorescence detection of Ace‐Tubulin and Tyr‐Tubulin, FGF20 was revealed to promote the polymerization of axon‐regenerated microtubules. Furthermore, FGF20 was also found to reduce the expression levels of necroptosis induced by SCI. These data suggest that FGF20 may exert a neuroprotective effect by inhibiting injury‐induced necroptosis, thereby facilitating functional recovery following SCI. Moreover, systemic administration of FGF20 holds promise as a potential therapeutic strategy for repairing the damaged spinal cord. The discovery paves the way for a novel avenue of growth factor research in the field of SCI.

## 1 INTRODUCTION

Spinal cord injury (SCI) is a neurological disorder that results in severe loss of motor and sensory function.^1^, ^2^ Neurons possess limited self‐repair capability after SCI due to their non‐dividing nature.^3^ The majority of SCI results from mechanical impact and can be pathophysiologically classified into primary tissue defects directly caused by the initial injury and secondary damage from factors such as autophagy, inflammation, ischaemia, oedema, oxidative stress and necroptosis.^4^, ^5^ Neurons are highly specialized cells that are difficult to regenerate.^6^ The natural self‐repair of spinal cord injuries is very limited.^7^ In SCI, a significant number of residual nerve cells undergo secondary damage following primary injury, leading to further functional impairment.^8^, ^9^ These secondary injuries will lead to a series of reactions such as cell apoptosis, tissue acidosis and free oxidative stress, which could promote the programmed necrosis of spinal cord neurons due to the damaged microenvironment, resulting in continuous impairment of spinal cord function.^10^, ^11^, ^12^ Therefore, restraining the cascade of secondary injury responses after SCI has emerged as a pivotal focus of treatment. Among these processes, necroptosis mediated by the mixed lineage kinase domain‐like proteins (MLKL) factor activated through receptor‐interacting protein kinases 1 (RIP1)/receptor‐interacting protein kinase 3 (RIP3) is a crucial mechanism underlying secondary functional deficits in SCI.

Necroptosis, also referred to as necrotic apoptosis, represents a distinct form of programmed cell death that differs from apoptosis.^13^, ^14^ It is Caspase‐independent cell death that is morphologically similar to necrosis through the release of injury‐related molecular patterns.^15^ Necroptosis exhibits analogous characteristics to conventional necrosis, such as disruption of the plasma membrane and induction of inflammatory reactions.^16^, ^17^ In the adult nervous system, compared to apoptosis, in the pathological state, Expression of death receptor family ligands sensitizes cells in the central nervous system to a modulated form called necrotic cell death mediated by RIP1, RIP3 and MLKL.^18^, ^19^ Necroptosis is associated with ischemic injury and neurodegenerative diseases, promoted further cell death and neuroinflammation in the pathogenesis of several neurodegenerative diseases,^20^, ^21^ including multiple sclerosis, amyotrophic lateral sclerosis, Parkinson's disease (PD) and Alzheimer disease, and can be triggered by extracellular stimuli that activate inflammation and cell death.^22^ The deregulation of necroptosis is also a key factor of many inflammatory diseases.^23^

The intracellular signalling pathways of necroptosis involve the formation of Complex IIb or necrosome, in which RIP1 binds and activates RIP3, regulating the expression of RIP3 and activating MLKL through phosphorylation, resulting in the translocation of MLKL to plasma and cytoplasmic membranes and the regulation of ion channel activity leading to necrosis.^24^, ^25^ In SCI, excessive or aberrant necroptosis can trigger a cascade of inflammatory responses by releasing damage‐associated molecular patterns (DAMPs), leading to detrimental pathological reactions such as immune damage caused by cytokine storms.^26^, ^27^, ^28^ Therefore, the inhibition of excessive necroptosis activation may represent a crucial strategy for safeguarding neuronal survival following SCI.^29^

Fibroblast growth factor 20 (FGF20), a member of the FGF family and a paracrine growth factor that is highly conserved in vertebrates, plays important roles in brain development and neuronal homeostasis.^30^ Human FGF20 exhibits a high degree of similarity to its murine counterpart, with an amino acid sequence identity approaching 95%.^31^, ^32^ Previous research has demonstrated that FGF20 is predominantly expressed within the central nervous system and possesses well‐established neurotrophic properties,^33^ which plays an important role in regulating the development and function of the central nervous system.^34^, ^35^ FGF20 enhances survival of cultured dopaminergic neurons,^36^ the beneficial impact of it in PD implies its potential neuroprotective effect in other disorders of the central nervous system.^37^ However, the neuroprotective effect of FGF20 in SCI and its underlying mechanisms remain unclear. We hypothesize that FGF20 may exert neuroprotective effects against cell death following SCI by attenuating excessive activation of necroptosis pathways.

In this study, the effects of FGF20 on mice with SCI were evaluated by oblique plane test, BMS (Basso Mouse Scale) and footprint analysis. The expression of neuron‐specific molecules and necrotic complexes such as MLKL, RIP1 and RIP3 was further examined. Our data show that FGF20 has a therapeutic effect on SCI in mice by inhibiting necrotic complex progression, and this study expands the current understanding of SCI research and the application of growth factors.

## 2 MATERIALS AND METHODS

### 2.1 Reagents and antibodies

Primary antibodies used in this study included neurofilament 200 (NF200, ab4680), glial fibrillary acidic protein (GFAP, ab7260), neuronal nuclei (NeuN, ab104224), growth‐associated protein 43 (GAP43, ab75810) and LC3‐II (ab192890). Secondary antibodies used were anti‐mouse antibody 488 (ab150113), anti‐rabbit antibody 488 (ab150077) and anti‐chicken antibody 488 (ab15090) from Abcam (MC, United Kingdom) and DAPI was also supplied by Abcam (MC, United Kingdom). Recombinant human fibroblast growth factor 20 (rhFGF20) was obtained from PeproTech (USA).

The secondary antibodies used were rabbit IgG conjugated with Alexa Fluor 488 (ab150061), Alexa Fluor 647 (ab150063) and Alexa Fluor 595 (ab150062). In addition, the secondary antibodies for western blot used rabbit HRP IgG. All of these were provided by Abcam (Cambridge, United Kingdom). The enhanced chemilluminescence kit was supplied by Bio‐Rad (Hercules, CA, USA).

### 2.2 Cell culture and treatment

Mouse pheochromocytoma PC12 cells (American culture system) were cultured in RPMI1640 basic medium (Gibco, USA) containing 10% fetal bovine serum (Karl's Bad, USA), 100 U/mL penicillin and 100 μg/mL streptomycin. PC12 cells were cultured in a 5% CO_2_ incubator at 37°C. Hydrogen peroxide (H_2_O_2_) was used to prepare the PC12 cell injury model, and untreated PC12 cells were used as a control group.

### 2.3 In vitro scratch assay

In vitro scratch assay was performed by linearly scratching the cells with the tip of a 200 μL pipette to create a cell‐free area. Citronellol was used as an inducer of necrotic apoptosis, while necrosulfonamide was utilized as an inhibitor of necrotic apoptosis. Following drug administration, cells were washed twice with PBS. The cell‐free area was photographed and analysed under an inverted microscope.

### 2.4 CCK‐8 assay

PC12 cells were seeded at a density of 10,000 cells per well in 96‐well plates. After the cells adhered to the plates, they were treated for 48 h with 1640 medium containing various doses of FGF20. After discarding the medium in the well plates, 90 μL serum‐free media and 10 μL CCK‐8 reagent were added to each well and incubated for 2 h at 37°C. Finally, the absorbance was detected at 450 nm.

### 2.5 Animal model of SCI

Forty adult female C57BL/6 mice were provided by the Jiangsu Jicui Yaokang Biotechnology Co., Ltd. The mouse had an average weight of 22–25 g at the time of surgery. The animal experiment was approved by the Animal Care and Use Committee of Wenzhou Medical University (wydw2022‐0387). The experiment was conducted in accordance with the guidelines for the care and use of laboratory animals of the National Institutes of Health. For the purpose of the experiment, these mice were randomly divided into three groups: a Sham group, a SCI group and an FGF20 treatment group.

After intraperitoneal injection of 1% pentobarbital sodium (6 mL/kg), the mice were cut along the middle of the back skin, exposing the 8th to 10th thoracic vertebrae. A moderate contusion was induced by clamping the T9 segment of the spinal cord for 5 s to produce an acute SCI model. The control group underwent the same surgical procedures, but the spinal cord was not injured. Animal care and treatment included bladder massage twice a day to help the mouse void urine.

### 2.6 Intraperitoneal injection of FGF20 to treat SCI

After establishing the SCI model, the treatment group mice were injected with 5 μg/kg FGF20 via intraperitoneal injection, and the injection was repeated every 2 days. The Sham surgery group was injected with physiological saline only. All animals were housed under SPF conditions and given the same moderate diet at fixed times. On the day of euthanasia, all animals were sacrificed humanely by intraperitoneal injection of 1% pentobarbital sodium (6 mL/kg) to obtain spinal cord tissue for subsequent experiments.

### 2.7 Locomotion recovery assessment

To assess the degree of recovery, two individuals who trained according to the standard scoring criteria monitored the physical condition and behaviour of the mouse. The BMS locomotor rating scale was used to evaluate hindlimb motor function, with a score range of 0–21. In the slope test, mice were placed on top of a slanted board, which is a rubber with the thickness of 6 mm, and the angle of inclination was measured. In the footprint test, the hind paws of the mouse were dipped in red dye for motion function analysis as described previously. The following parameters were used to evaluate motor function: (1) weight support, (2) leg extension spasm, (3) number of steps and (4) foot posture.^38^, ^39^

### 2.8 Haematoxylin and eosin staining and Nissl staining

Spinal cord paraffin sections were performed with haematoxylin and eosin and immunohistochemistry staining. The slides were incubated in 1% methyl violet for Nissl staining, then observed and photographed under a light microscope to observe the overall recovery effect of spinal cord.

### 2.9 Immunofluorescence staining

Immunofluorescence staining was performed at room temperature. Tissue sections were treated with 5% bovine serum albumin in PBS containing 0.1% Triton X‐100 for 1 h. Then, the sections were incubated with different primary antibodies, including NeuN (1:300), GAP43 (1:300), NF200 (1:300), Tyr‐tubulin (1:300), Ace‐Tubulin (1:300), GFAP (1:300), P‐MLKL (1:300), P‐RIP1(1:300) and P‐RIP3(1:300) in the same buffer at 4°C overnight. After that, corresponding secondary antibodies were used and incubated at 37°C for 1 h. Finally, the cell nuclei were stained with DAPI. Images were captured using a Nikon microscope (Nikon, Tokyo, Japan), and the results were analysed by ImageJ software (version 1.8.0). The cell count was determined using ImageJ, which also facilitated the calculation of fluorescence intensity within the delineated target cells. Images were captured from three samples per group. Statistical graphs were created using Prism 7 (GraphPad, San Diego, CA, USA), and the data analysis was conducted with Excel and GraphPad Prism 7. Results are presented as the mean ± standard deviation (SD), with a *p* value of less than 0.05 indicating statistical significance.

### 2.10 Western blot

Tissue samples from the central part of the SCI area were obtained and frozen at −80°C for subsequent protein blot analysis. Cell lysates containing PMSF and phosphatase inhibitors were homogenized, and the supernatant was collected after centrifugation. The protein concentration was quantified using the Coomassie brilliant blue method. A 12.5% gel was used for electrophoresis, and each lane was loaded with 40 μg of protein. The proteins were then transferred onto a PVDF membrane (Bio‐Rad, Hercules, CA, USA). The membrane was incubated with rapid block buff (Beyotime, Shanghai, China) for 20 min, followed by overnight incubation with primary antibodies at 4°C, the type of primary antibodies including P‐MLKL (1:1000), MLKL (1:1000), P‐RIP1(1:1000), RIP1(1:1000), P‐RIP3(1:1000), RIP3 (1:1000) and GAPDH (1:5000). After that, corresponding secondary antibodies were used and incubated at room temperature for 2 h. The signal was detected using a ChemiDoc XRS+ imaging system (Bio‐Rad). Band densities were measured and quantified using ImageJ software.

### 2.11 Statistical analysis

Statistically, data are expressed as the mean ± SEM. For two experimental groups, Student's *t*‐test was used to determine statistical significance. *p* < 0.05 were considered significant. For more than two experimental groups, one‐way analysis of variance (anova) and Dunnett's post hoc test were used for statistical evaluation, and *p* < 0.05 were considered significant.

## 3 RESULTS

### 3.1 FGF20 enhances motor function recovery after SCI

**FIGURE 1:**
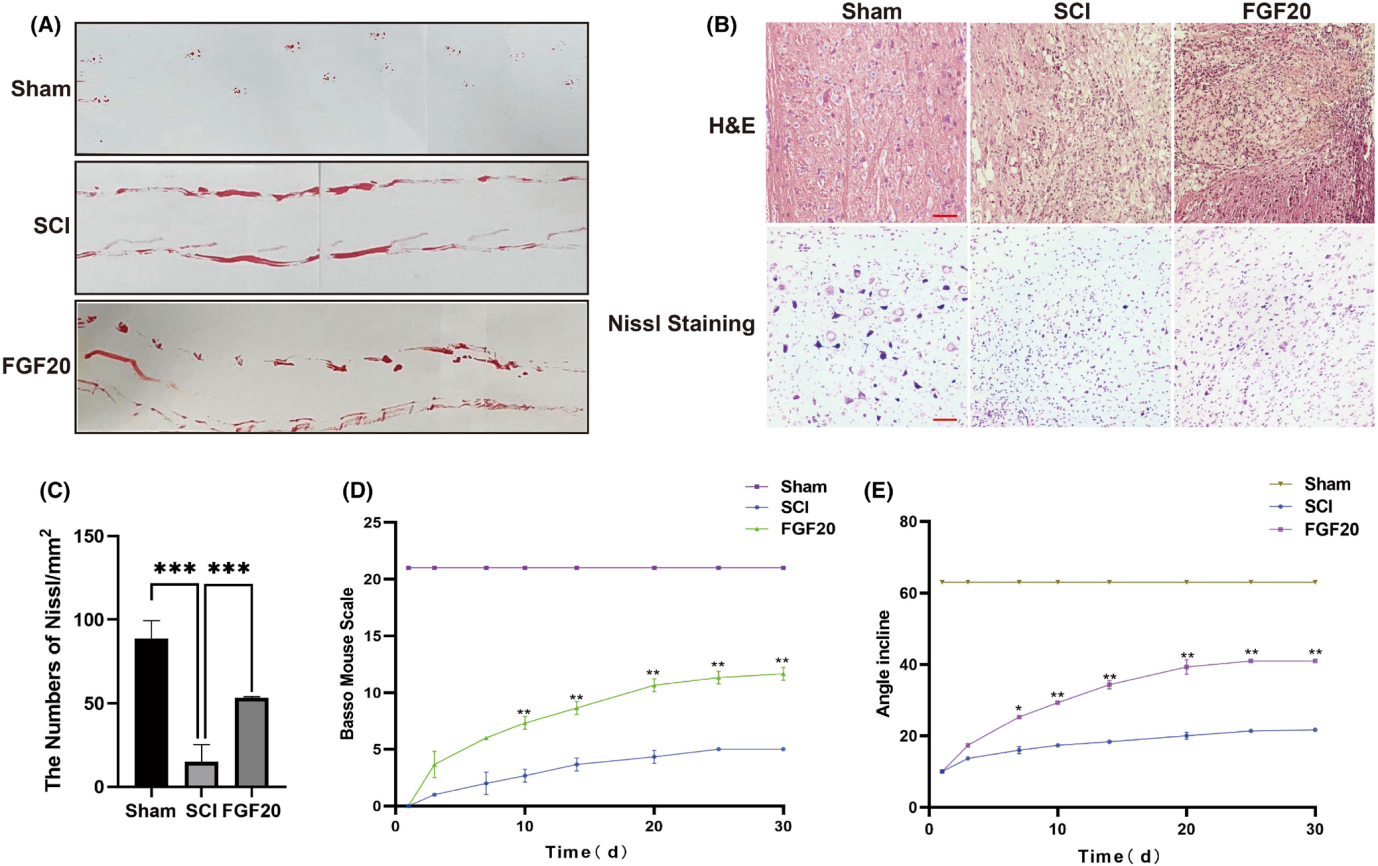
FGF20 promotes motor function recovery after spinal cord injury in mice. (A) Footprint analyses of Sham group, SCI group and FGF20 group. (B) Haematoxylin and eosin staining and Nissl staining images for the Sham group, SCI group, FGF20 group. Scale bar: 10 μm. (C) Analysis of the Nissl staining results. ***p < 0.01 versus SCI group. Data are the mean values ± SEM, *n* = 3. (D) The BMS scores of the mice in the Sham, SCI and FGF20 groups. **p < 0.01 versus the SCI group with statistical significance. Data are represented as mean ± SD (*n* = 3). (E) The inclined plane test scores of mice in the Sham, SCI and FGF20 groups. *p < 0.05, **p < 0.01 versus the SCI group with statistical significance. Data are represented as mean ± SD (*n* = 3).

The motor ability of mouse was evaluated using the BMS and the inclined plane test scores. The FGF20 group showed higher scores than other treatment groups. Further, in the footstep imprinting experiment, the Sham group exhibited clear and natural footprints, while the mice in SCI group showed severe imprinting disorder with a wave shape and dragged movement. The footprints of mice in the FGF20 treatment group improved, and the footprint test analysis showed further improve of hind limb coordination and reduced toe dragging. In contrast, footprints obtained from SCI group displayed inconsistent gait and extensive toe dragging, as showed by ink traces from the hind limbs of mice (Figure 1A,D,E).

### 3.2 FGF20 confers neuroprotection against necroptosis and facilitates axonal penetrate the glial scar

**FIGURE 2:**
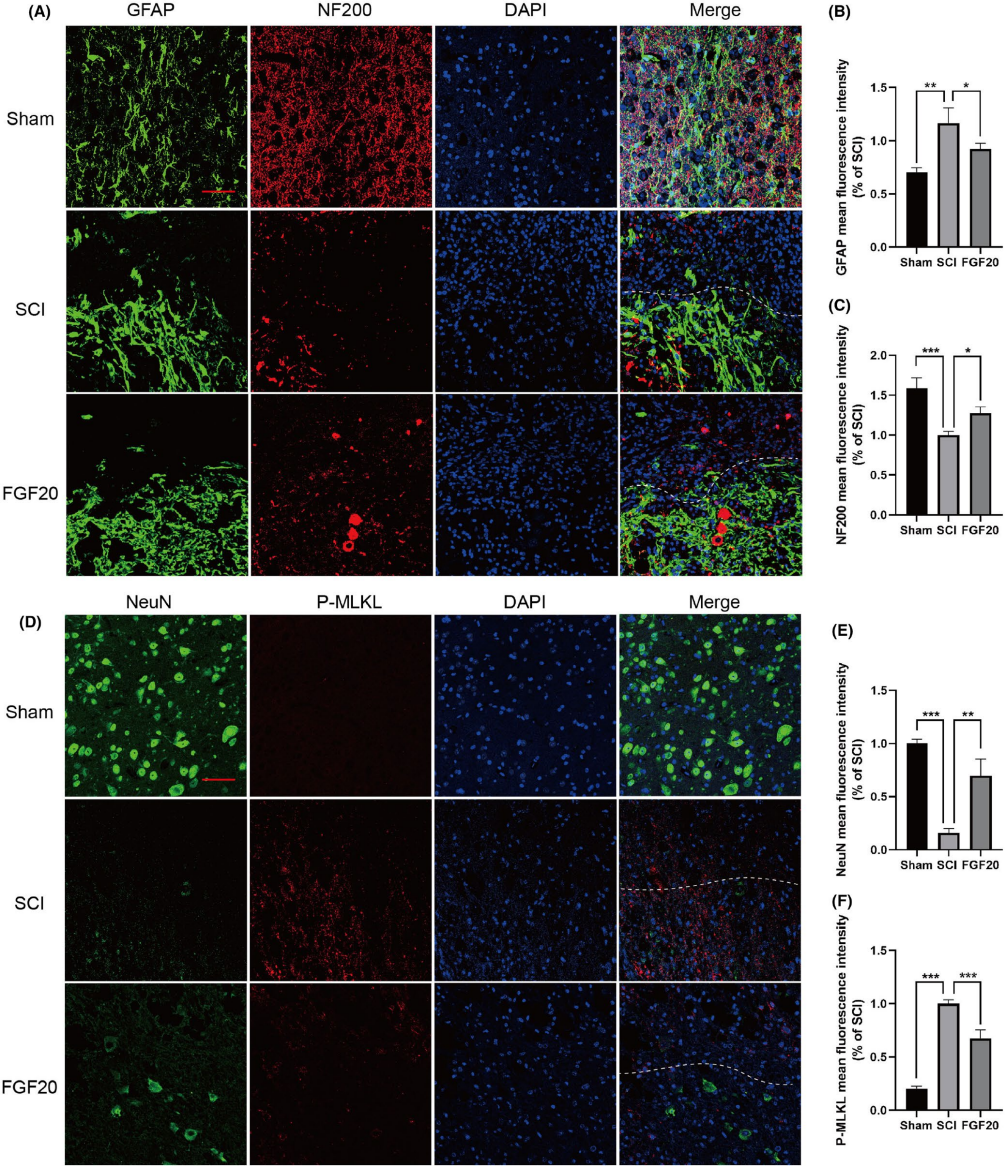
FGF20 protects neuronal necrosis in mouse after spinal cord injury, reduces scar formation and promotes neurofilament regeneration. (A) Immunofluorescence staining of NF200 (bright red) and GFAP (bright green). The nuclei are labelled by DAPI (blue), scale bar: 100 μm. (B) Immunofluorescence staining of NeuN (bright green) and P‐MLKL (bright red). The nuclei are labelled by DAPI (blue), scale bar: 100 μm. (B) Statistical analysis of GFAP expression in each group. (C) Statistical analysis of NF200 expression in each group. (E) Statistical analysis of NeuN expression in each group. (D) Statistical analysis of P‐MLKL expression in each group. *p < 0.05, **p < 0.01 and ***p < 0.001 versus the SCI group with statistical significance. Data are represented as mean ± SD (*n* = 3).

Co‐staining of NF200 and GFAP showed an increase in NF200 expression after FGF20 treatment (Figure 2A–C), indicating enhanced growth and regeneration of nerve fibres and penetrating the scar tissue. Fluorescence co‐staining of NeuN and P‐MLKL after SCI revealed more red fluorescence (necrosis marker) at the edge and center of injured spinal cord (Figure 2D–F), indicating neuronal necrosis and cell membrane rupture. Compared to the SCI group, FGF20‐treated mouse exhibited a significant increase in NeuN fluorescence intensity and a higher survival rate of neurons. These results suggest that FGF20 has neuroprotective and neuroregulatory functions in SCI mice.

### 3.3 The addition of FGF20 provides neuroprotection by preserving neuronal protein expression and enhancing microtubule polymerization

**FIGURE 3:**
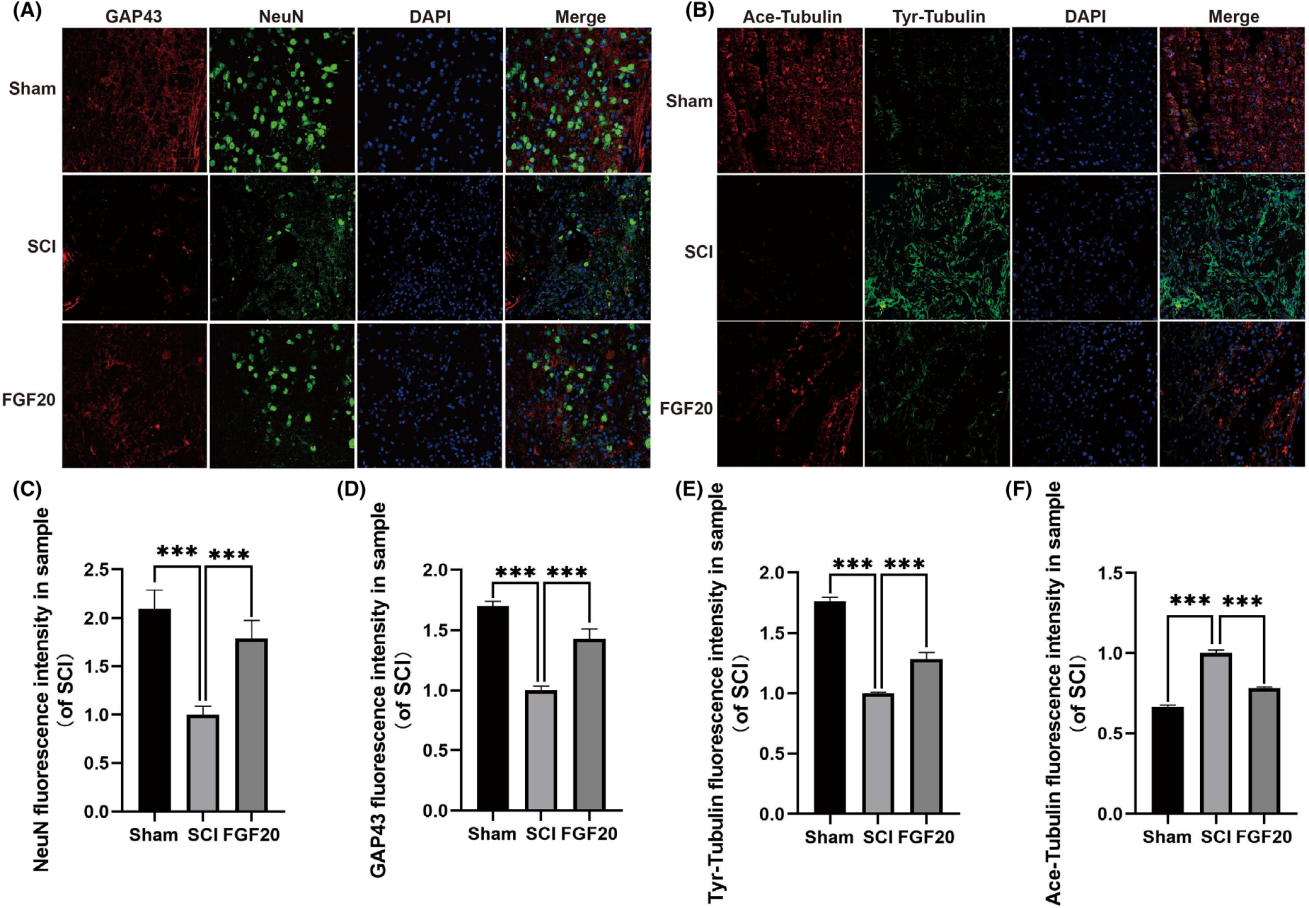
FGF20 enhances neuronal microtubule aggregation and promotes axonal regeneration in mouse after spinal cord injury. (A) Immunofluorescence staining of GAP43 (bright red) and NeuN (bright green). The nuclei are labelled by DAPI (blue), scale bar: 100 μm. (B) Immunofluorescence staining of Ace‐Tubulin (bright red) and Tyr‐Tubulin (bright green). The nuclei are labelled by DAPI (blue) and the magnification is ×40. (C–F) Statistical analysis of GAP43, NeuN, Ace‐Tubulin and Tyr‐Tubulin expression in each group. ***p < 0.001 versus the SCI group with statistical significance. Data are represented as mean ± SD (*n* = 3).

The survival of neurons after SCI is important for the recovery of motor function, and the protective effect of FGF20 on neurons in SCI mouse was evaluated by staining with NeuN protein. Immunofluorescence staining showed that the survival rate of neurons in the FGF20‐treated group was higher than that in the SCI group, and also promoted the expression of GAP43 (Figure 3A,C,D). The co‐expression of Tyr‐tubulin and Ace‐tubulin further confirmed that the necrosis of neurons in the injury area was inhibited in the FGF20 group (Figure 3B,E,F). The increased expression of GAP43 and Ace‐tubulin indicated that FGF20 can protect the survival of neurons after injury and maintain stability of microtubules, suggesting that FGF20 plays a role in promoting the repair of damaged neurons. These results suggest that FGF20 exerts neuroprotective and reparative effects on mice with SCI by upregulating the expression of NeuN, GAP43 and Ace‐tubulin.

### 3.4 FGF20 reduces the level of P‐MLKL/ P‐RIP1/p‐RIP3 necroptotic complex formation in vivo and vitro

**FIGURE 4:**
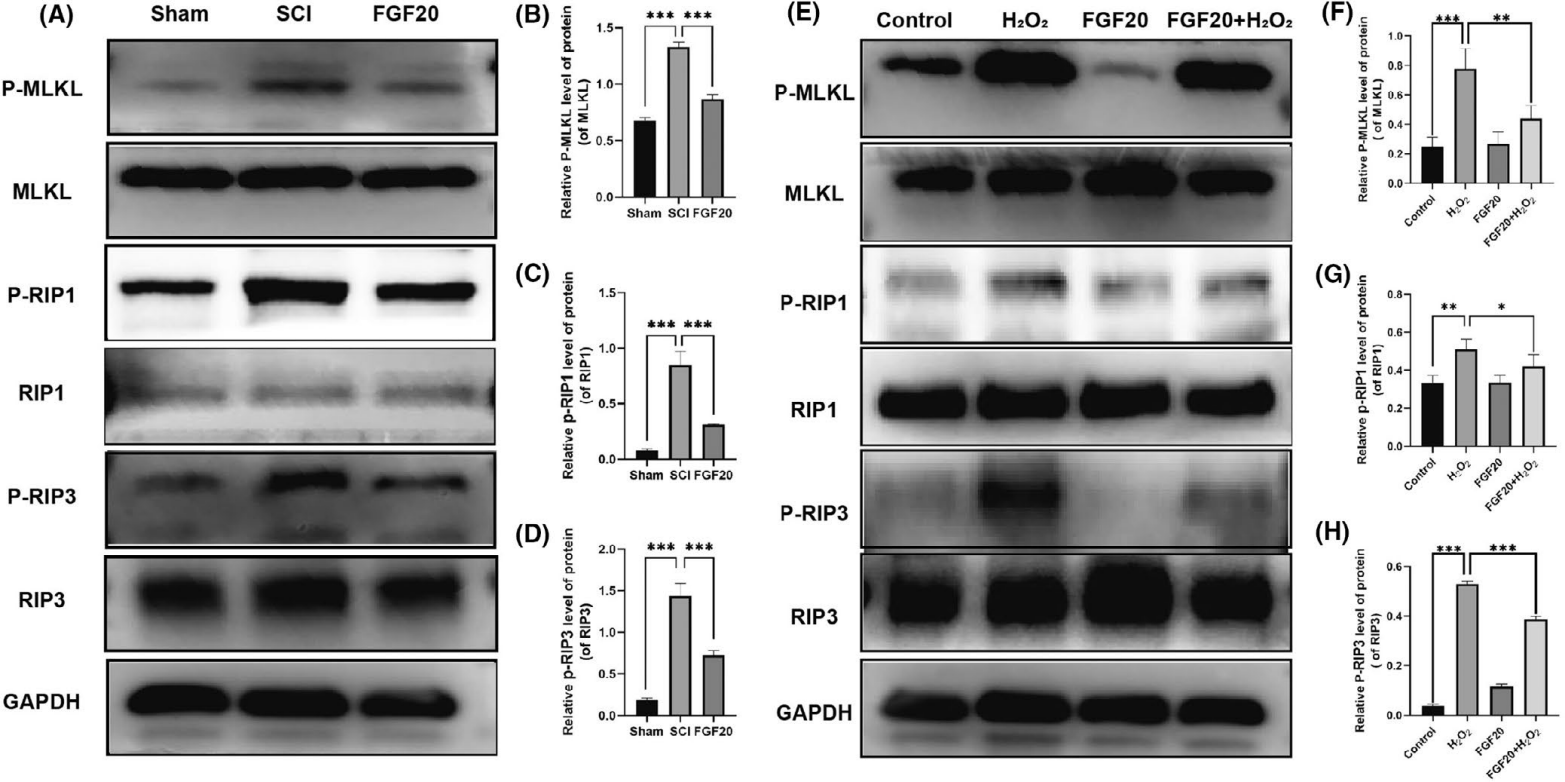
FGF20 inhibits H_2_O_2_ induced programmed necrosis complex formation in vitro and vivo. (A) Western blotting analysis showing protein expressions of MLKL, P‐MLKL, RIP1, P‐RIP1, RIP3, P‐RIP3 and GAPDH is used as a loading control. (B–D) Quantitative analysis of P‐MLKL, P‐RIP1 and p‐RIP3 protein expression. (E) Western blot analysis showing the protein expression of MLKL, P‐MLKL, RIP1, P‐RIP1, RIP3, P‐RIP3 and GAPDH in the Sham group, SCI group and FGF20 group. GAPDH is used as a loading control. (F–H) Quantitative analysis of p‐MLKL, p‐RIP1 and p‐RIP3 protein expression.*p < 0.05, **p < 0.01 and ***p < 0.001. Data are expressed as the mean values ± SD (*n* = 3).

The utilization of FGF20 impedes the formation of necroptotic bodies in animals with spinal cord injuries. Western blot experiment was used to measure the expression of necrotic complex related indicators ex vivo. Western blot analysis showed no significant difference in non‐phosphorylated MLKL, RIP1 and RIP3 between the groups. Compared with the SCI group, the FGF20 group showed decreased activation levels of the necroptotic complex core proteins MLKL, RIP1 and RIP3, manifested by decreased expression of P‐MLKL, P‐RIP1 and P‐RIP3 (Figure 4A–D). FGF20 can inhibit the cell necrosis process of PC12 cells. To observe the expression of necroptotic complexes induced by programmed cell death after FGF20 treatment, Western blot analysis was performed. We first demonstrated that FGF20 can be expressed in the nervous system after administration(Figure S2). We found that the expression of the necroptotic complex core proteins MLKL, RIP1 and RIP3 was stable in the inactive state, but they were phosphorylated after H_2_O_2_‐induced injury with the expression of P‐MLKL, P‐RIP1 and P‐RIP3 increased synchronously (Figure 4E). FGF20 treatment decreased the expression of P‐MLKL, P‐RIP1 and P‐RIP3, resulting in a decrease in the expression level of the cell necroptotic complex (Figure 4F–H). We also supplemented the effect of FGF20 on inflammation in animals, but the results showed that the effect of FGF20 on inflammation was not significant (Figure S1).These data suggest that FGF20 has the ability to inhibit the formation of necroptotic complexes to reduce cell necrosis. These findings suggest that FGF20 exerts an inhibitory effect on the expression of the necroptotic complex core while enhancing the expression of necrosis‐related inhibitory proteins, ultimately reducing cell necrosis.

### 3.5 FGF20 has a protective effect on PC12 cells stimulated by H_2_O_2_

**FIGURE 5:**
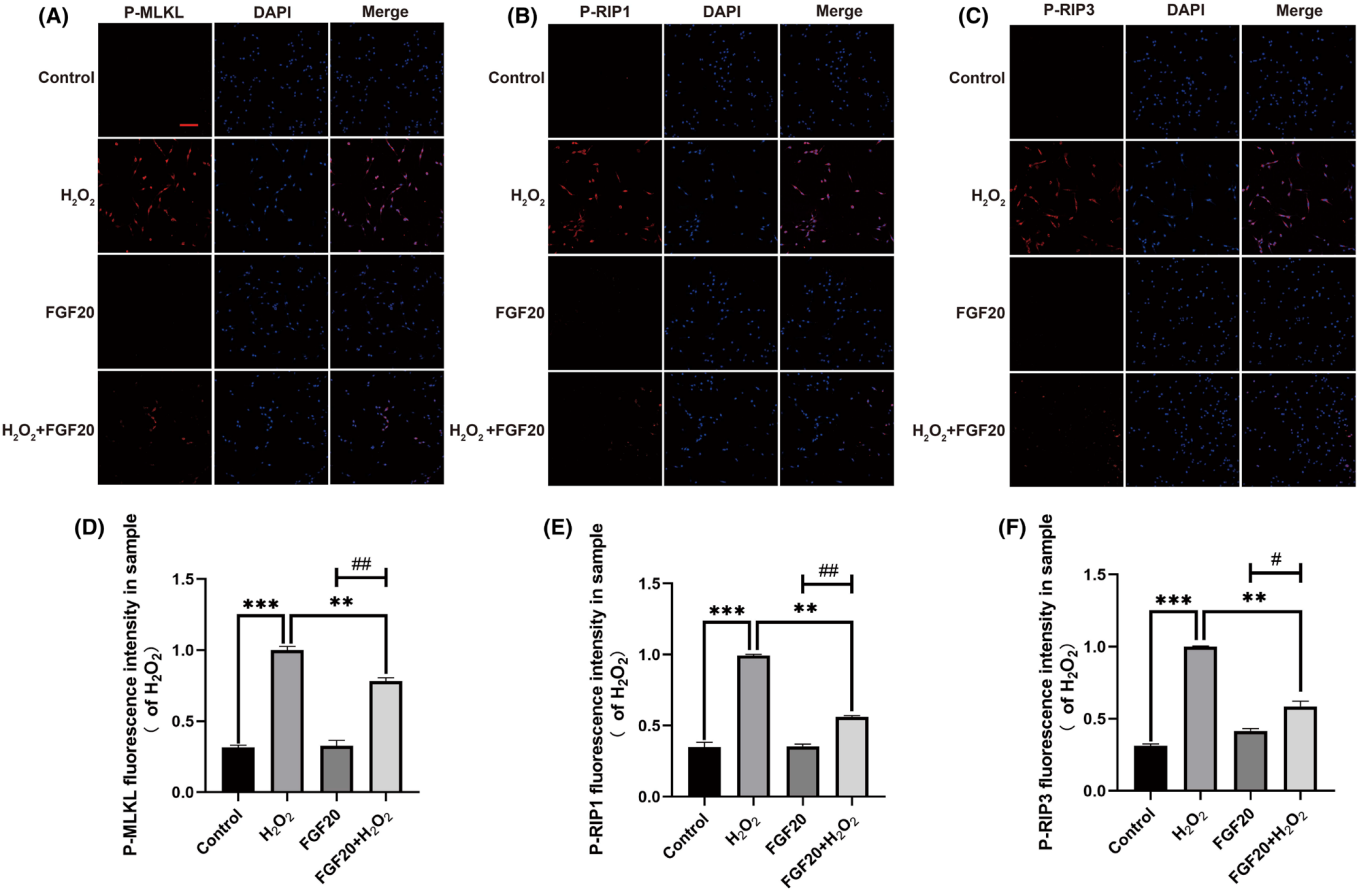
FGF20 protects PC‐12 cells from H_2_O_2_ damage. (A–C) Immunofluorescence staining of P‐MLKL, P‐RIP1 and P‐RIP3 (bright red), in the PC‐12 group, PC‐12 + H_2_O_2_ group, PC‐12 + FGF20 group and PC‐12 + FGF20 + H_2_O_2_ group. The nuclei are labelled by DAPI (blue) and magnification is ×40. Scale bar: 50 μm. (D–F) Mean fluorescence intensity of P‐MLKL, P‐RIP1 and P‐RIP3 in different groups (% of H_2_O_2_), **p < 0.01, ***p < 0.001 versus the H_2_O_2_ group. #*p* < 0.05, ##*p* < 0.01 versus the FGF20 group. Data are represented as mean ± SD (*n* = 3).

It has been reported that exposure of PC‐12 cells to H_2_O_2_ can alter cell morphology and induce programmed cell death. In this experiment, we hypothesized that pretreatment with FGF20 would protect cell viability and reverse H_2_O_2_‐induced cell death by inhibiting necrotic apoptosis. Immunofluorescence results showed that the expression of P‐MLKL, P‐RIP1 and P‐RIP3 (red fluorescence) increased in PC12 cells treated with H_2_O_2_, indicating that PC‐12 cells underwent necroptosis (Figure 5A–C). In contrast, the expression of P‐MLKL, P‐RIP1 and P‐RIP3 significantly decreased after FGF20 treatment, indicating that FGF20 can protect PC12 cells from H_2_O_2_‐induced cell death by inhibiting necrotic apoptosis. Western blot results showed similar results, with the expression of P‐MLKL, P‐RIP1 and P‐RIP3 upregulated in cells treated with H_2_O_2_, and downregulated after FGF20 treatment (Figure 4E–H). In summary, our results indicate that FGF20 can protect PC‐12 cells from H_2_O_2_‐induced cell death by inhibiting the process of necroptosis.

### 3.6 FGF20 promotes cell proliferation and migration

**FIGURE 6:**
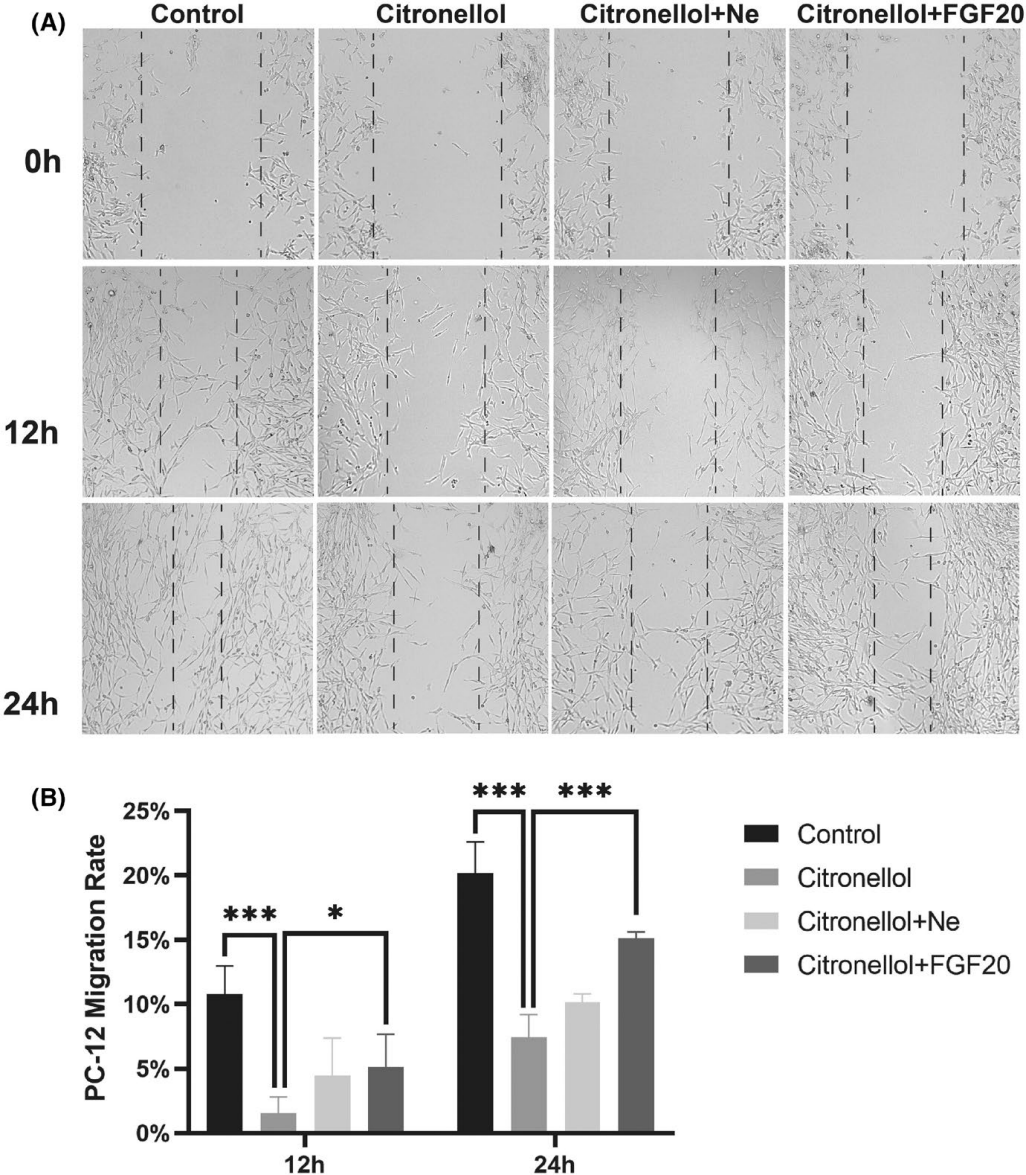
FGF20 can promote the migration rate of PC12 cells. (A) Scratch healing of PC‐12 group, PC‐12 + Citronellol group, PC‐12 + Necrosulfonamide + Citronellol group and PC12 + FGF20 + Citronellol group. All images were taken with an inverted phase contrast microscope at 0, 12 and 24 h after cell scratches. Scale bar: 10 μm. (B) Cell mobility statistics of PC‐12 group, PC‐12 + Citronellol group, PC‐12 + Necrosulfonamide + Citronellol group and PC12 + FGF20 + Citronellol group. A two‐way analysis of variance (ANOVA) test was used to analyse cell migration results, and then Dunnett's post hoc test was used. In the 12 h group, *p < 0.05 versus the PC‐12 + Citronellol group. In the 24 h group, *p < 0.05, ***p < 0.01 versus the PC‐12 + Citronellol group. Data are represented as mean ± SD (*n* = 3).

PC12 cells have been shown to have similar functions to normal neuronal cells, such as cell morphology and physiological activities. We investigated whether FGF20 could promote cell migration and repair by inhibiting the process of necrotic apoptosis. Citronellol was used to activate the MLKL necroptotic complex in PC12 cells, and it was found that FGF20 protected against Citronellol‐induced program cell death in a time‐dependent manner, promoting cell migration and wound repair (Figure 6A,B). As a positive control, it was also found that Necrosulfonamide protected against cell migration inhibition by inhibiting the Citronellol‐induced program cell death process (Figure 6A,B). FGF20 is better than Necrosulfonamide in promoting cell mobility. The results of the CCK8 assay were similar. FGF20 at low concentration could promote cell proliferation (Figure S3).

To observe the protective effect of FGF20 on neurons, haematoxylin and eosin staining and Nissl staining were performed on longitudinal sections of the spinal cord in each group. Compared to the Sham group, the spinal tissue section of SCI group at Day 30 showed a significant increase in cavitation and disordered structure, which indicates the aggravation in injury. After FGF20 administration, significant protective activity was observed with fewer necrotic, and fewer nuclear condensed in neuron cells. The survival rate of swollen neurons in the SCI group decreased, and Nissl staining showed that the morphology of the cells was blurred, which was restored after FGF20 treatment (Figure 1B,C). In conclusion, the number of neurons in the FGF20 treatment group was significantly higher than that in the SCI group, indicating the neuroprotective effect of FGF20 on SCI mice.

## 4 DISCUSSION

The secondary injury that ensues after SCI is the primary cause of extensive neuronal death and impaired motor function recovery.^40^, ^41^ Due to the challenging nature of nerve cell recovery,^42^ safeguarding these cells is the most crucial factor in promoting SCI rehabilitation.^43^ Our study showed that FGF20 administration could inhibit injury‐induced necroptosis and protect neuronal survival, thereby promoting local neurofilament growth, effectively promoting the recovery of SCI in mice and restoring hindlimb motor function, indicating for the first time that FGF20 administration has good potential in SCI repair therapy.

In recent years, necroptosis‐mediated programmed cell death has garnered significant attention as a novel and finely regulated mechanism of cellular demise. Necroptosis, an important programmed cell death mechanism, serves as a vital regulator in disease.^20^, ^23^, ^44^ The key step under the necroptosis pathway induced by TNF‐α is the kinase‐regulated process of RIP1‐RIP3‐MLKL.^45^, ^46^ After cellular necrosis occurs, the combination of RIP1 and RIP3 leads to the activation of RIP3's kinase activity.^47^ RIP3 then undergoes autophosphorylation, enabling its specific binding to substrate MLKL, which is subsequently phosphorylated by RIP3.^48^ The RIP1/RIP3/MLKL at this stage becomes activated, transmitting a death signal downstream and ultimately leading to necrotic cell death.^49^, ^50^, ^51^ The phosphorylation of these molecules not only facilitates the formation of detrimental necrotic bodies, but also initiates multiple cascades that mediate various pathological processes and result in subsequent damage.^49^ A growing number of cell types in different pathophysiological microenvironments have been shown to have necroticptosis, such as senescence,^52^ amyotrophic lateral sclerosis and Alzheimer's disease and autoimmune diseases (multiple sclerosis).^53^ This study found that FGF20 can effectively inhibit the expression of P‐MLKL/P‐RIP1/P‐RIP3 in SCI, reduce the initiation of necroptosis, thereby reducing the loss of early neurons caused by injury and promoting neuronal regeneration, which may provide a promising and reasonable strategy for the treatment of SCI.

FGF20 has been found to be an endogenous neurotrophic factor for dopaminergic neurons, which can enhance the survival of midbrain dopaminergic neurons and has important clinical application value in the treatment of degenerative neurological diseases such as PD.^54^, ^55^, ^56^ It has also been reported that FGF20 can prevent BBB disruption after traumatic brain injury by upregulating the expression of junctional proteins and inhibiting inflammatory responses.^36^, ^57^ ‘Anthony et al.^58^ reported that FGF gene expression levels are decreased when necroptosis is induced, while Lin et al.^26^ reported that FGF‐20 protein levels are increased when necroptosis occurs. There is no direct evidence that FGF is effective in regulating necroptosis’. FGF‐20 is a potent pro‐proliferation FGF, and its effect in promoting cell proliferation is greater than its effect in regulating cell metabolism. After SCI, the blood supply of the spinal cord is deficient and it is difficult to supply nutrients effectively.^59^, ^60^ Under the powerful effect of FGF‐20 on proliferation, the neurons in the injured area may have difficulty in competing for nutrients, which leads to the aggravation of necrotizing apoptosis. The growth factors at the end of the FGF family have strong metabolic regulation ability, but their ability to promote proliferation is weak.^61^, ^62^ Therefore, FGF20 used in this study mainly takes advantage of its metabolic regulation ability to regulate microtubule stabilization and inhibit the occurrence of necroptosis in neurons.^63^, ^64^

In summary, FGF20 plays a neuroprotective role by inhibiting injury‐induced necroptosis, thereby promoting functional recovery after SCI. Collectively, systemic application of FGF20 is expected to be a potential therapeutic strategy for repairing injured spinal cord.

## AUTHOR CONTRIBUTIONS

**Sipin Zhu:** Funding acquisition (equal); resources (equal); supervision (equal). **Xiong Cai:** Conceptualization (equal); data curation (equal); formal analysis (equal). **Zhenwen Xie:** Formal analysis (equal). **Juan Zhao:** Data curation (equal). **Wenjie Lu:** Conceptualization (equal). **Zhongwei Zhu:** Formal analysis (equal). **Min Chen:** Formal analysis (equal). **Zhiyang Huang:** Conceptualization (equal); writing – original draft (equal); writing – review and editing (equal). **Yibo Ying:** Validation (equal); visualization (equal). **Yining Fu:** Software (equal). **Jiake Xu:** Data curation (equal); supervision (equal).

## CONFLICT OF INTEREST STATEMENT

The authors declare no conflict of interest.

## Supporting information


Figure S1.

## Data Availability

The data that support the findings of this study are available from the corresponding author upon reasonable request.
