# Supplementary material for: FGF20 promotes spinal cord injury repair by inhibiting the formation of necrotic corpuscle P‐MLKL/P‐RIP1/P‐RIP3 in neurons
Source: J Cell Mol Med. 2024 Dec 16;28(24):e70109. doi: 10.1111/jcmm.70109 (PMC11647335; doi:10.1111/jcmm.70109)
Supplement: Supplementary file 1 — Figure S1. [file JCMM-28-e70109-s001.docx]

Supplementary Materials for

**FGF20 promotes spinal cord injury repair by inhibiting the formation of necrotic corpuscle P-MLKL/P-RIP1/P-RIP3 in neurons**

*Corresponding author. Email: [sipinzhu@163.com](mailto:sipinzhu@163.com)

**This PDF file includes:**

Figs. S1 to S3

**
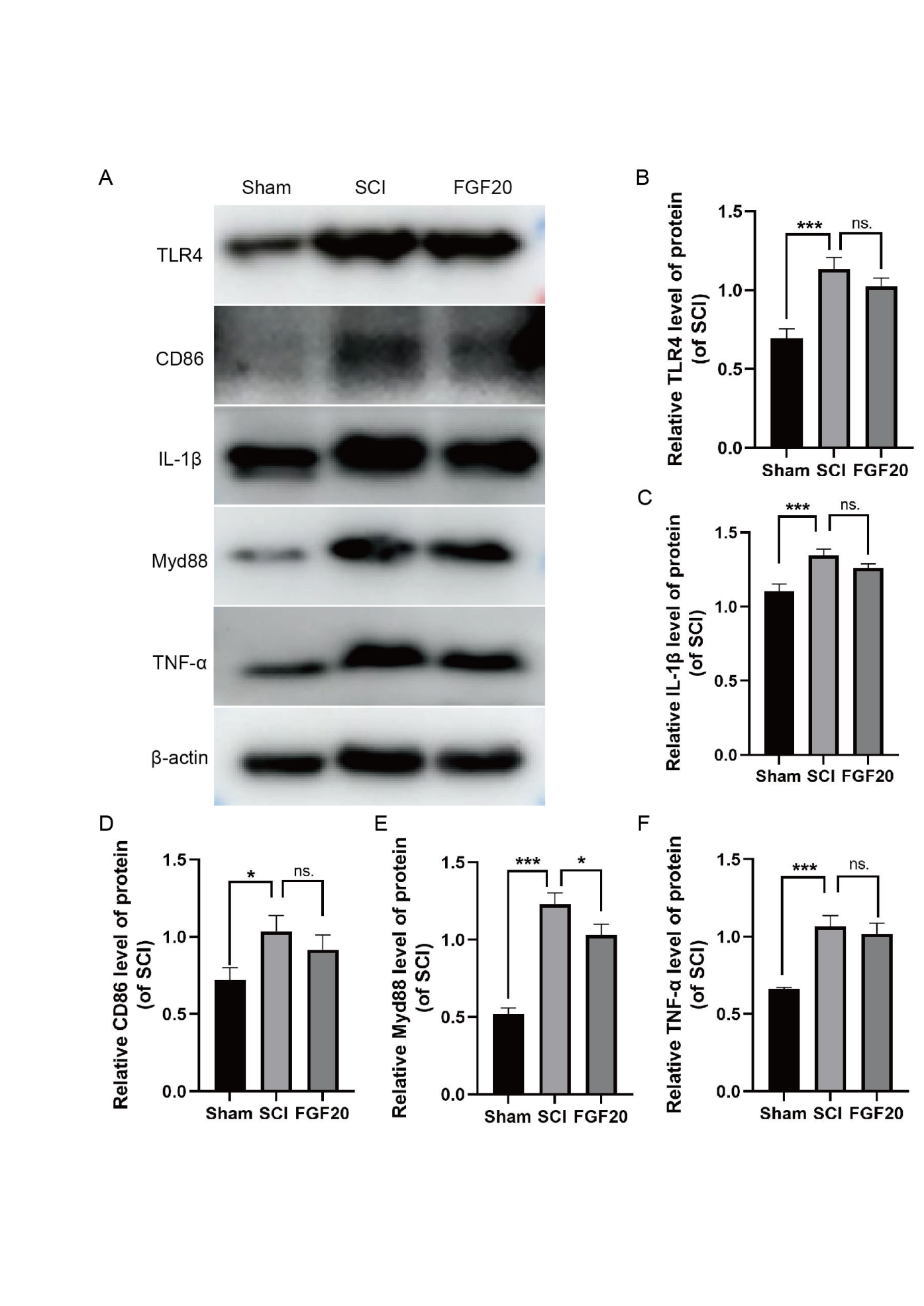
**

**SFigure 1. FGF20 inhibits H2O2 induced programmed necrosis complex formation in vitro and vivo**

1. Western blot analysis showing the protein expression of TLR4, CD86, IL-1β, Myd88, TNF-α, and β-actin in the Sham group, SCI group, and FGF20 group. β-actin is used as a loading control. (B-F) Quantitative analysis of p-TLR4, CD86, IL-1β, Myd88, TNF-αprotein expression.* represents P<0.05, ** represents P<0.01 and *** represents P<0.001. Data are expressed as the mean values ± SD (n=3).

**
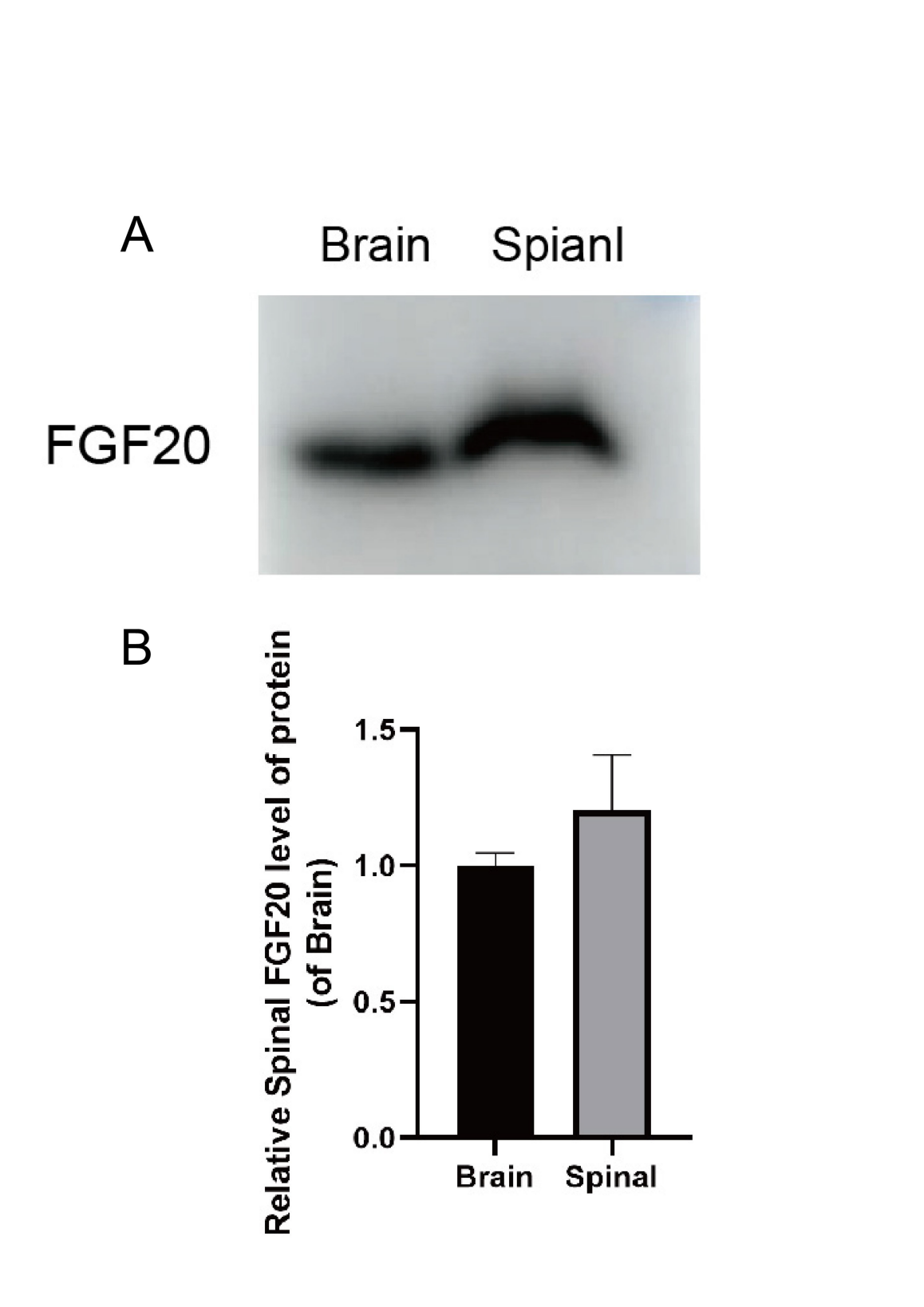
**

**SFigure 2. Expression of FGF20 protein in brain and spinal cord**

1. Western blot analysis showing the protein expression of FGF20 in the SCI group. (B) Quantitative analysis of FGF20 protein expression.Data are expressed as the mean values ± SD (n=3).

**
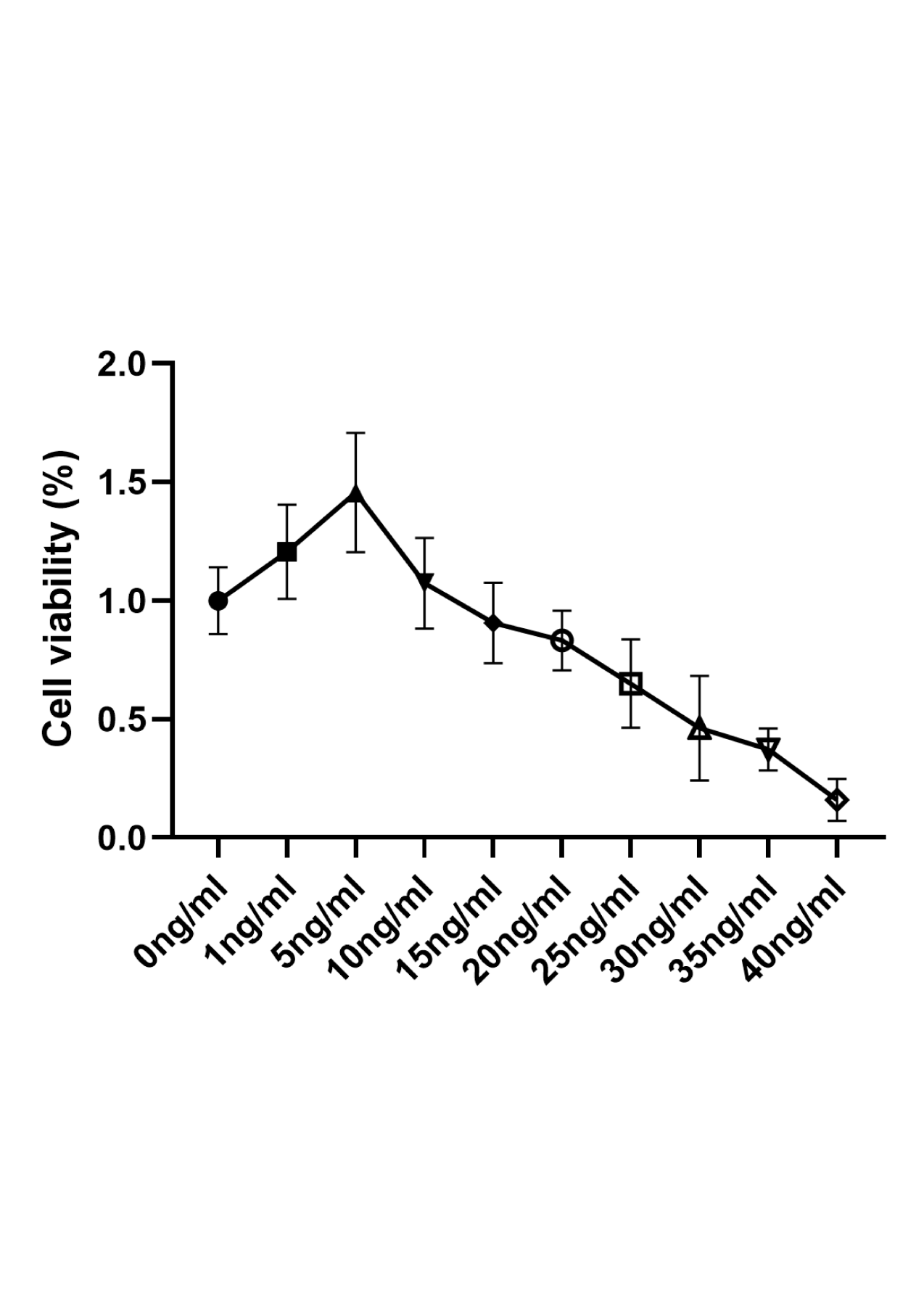
**

**SFigure 3. Relative cell viability of PC12 cells cultured with FGF20 at different concentrations for 24 h.**
